# Supplementary figures and images for: Dynamic coupling of residues within proteins as a mechanistic foundation of many enigmatic pathogenic missense variants
Source: PLoS Comput Biol. 2022 Apr 7;18(4):e1010006. doi: 10.1371/journal.pcbi.1010006 (PMC9017885; doi:10.1371/journal.pcbi.1010006)

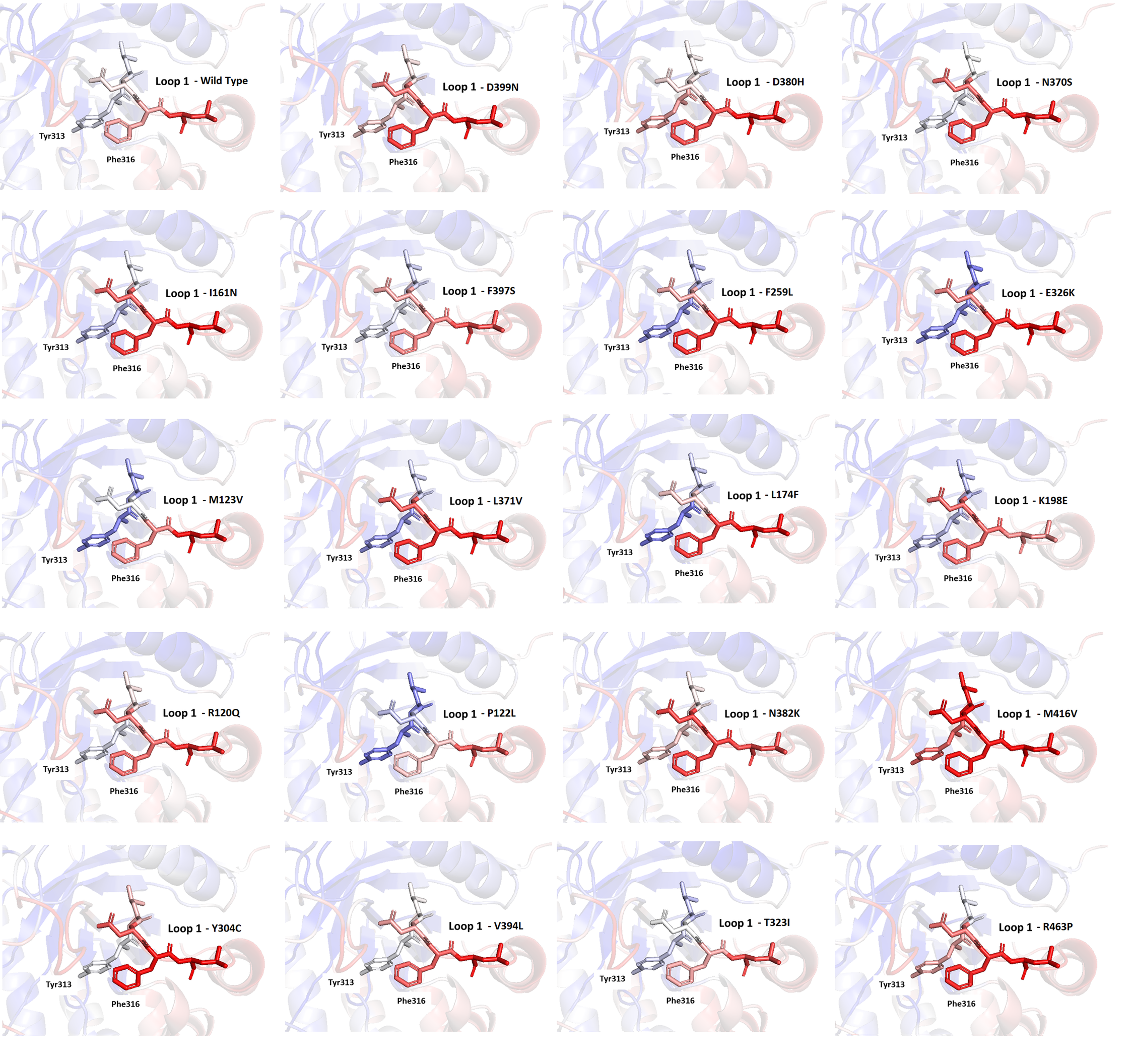

Supplement: S1 Fig — DFI here is a color code within a spectrum of red-white-blue where red shows the highest, and blue shows the lowest flexible sites. (TIF) [file pcbi.1010006.s001.tif]

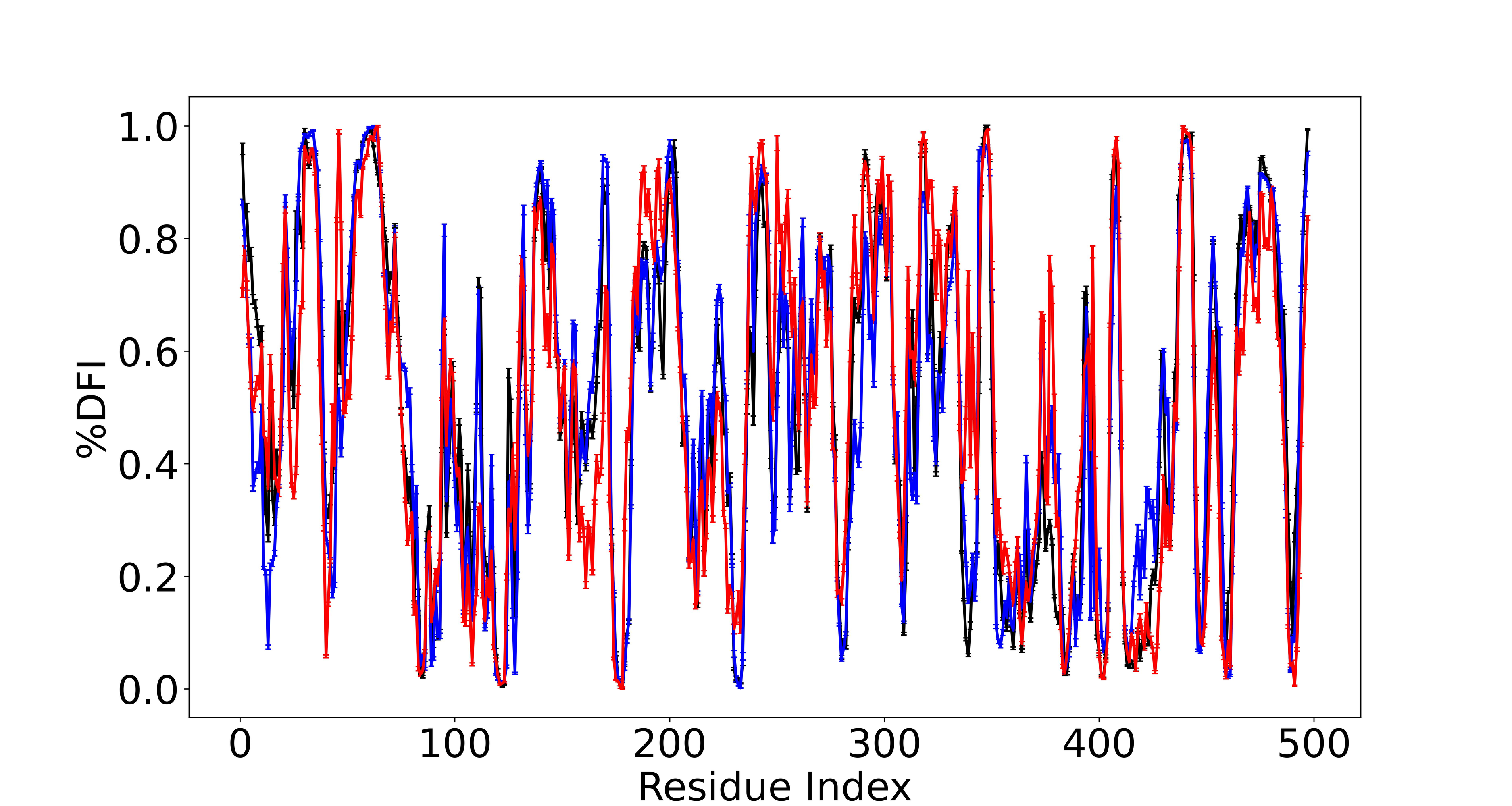

Supplement: S2 Fig — Black: average %DFI values calculated using covariance matrix data over 400ns to 600ns of the wild type GCase simulation. Blue: average %DFI values calculated using covariance matrix data over 600ns to 800ns of the wild type GCase simulation. Red: average %DFI values calculated using covariance matrix data over 800ns to 1ms of the wild type GCase simulation. All profiles use 50 ns moving windows that overlap by 25 ns when calculating average %DFI. (TIF) [file pcbi.1010006.s002.tif]

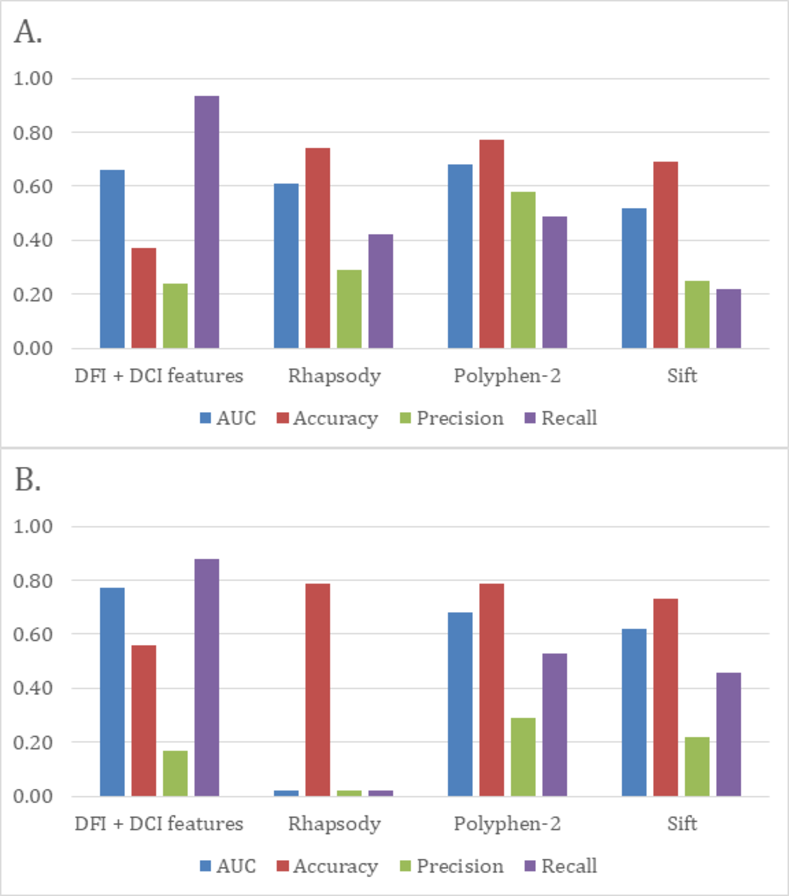

Supplement: S3 Fig — Bar plots showing accuracy, precision, recall, and area-under-the-curve (auc) values for four different methods including our DFI + DCI features. (A) The prediction methods were evaluated using only fast evolving sits according to ConSurf. (B) The prediction methods were evaluated using only fast evolving sits according to MEGA. Note that for our highly evolving subset, rhapsody returned 0 true positive and 0 false negative values, causing AUC, precision, and recall to be either zero or incalculable. Using either set of highly evolving sites, we are slightly better in AUC and comparable in precision. However, the dynamics based classifier have slightly lower values for accuracy owing to higher false positive rates. (TIF) [file pcbi.1010006.s003.tif]
