# Supplementary material for: Dynamic coupling of residues within proteins as a mechanistic foundation of many enigmatic pathogenic missense variants
Source: PLoS Comput Biol. 2022 Apr 7;18(4):e1010006. doi: 10.1371/journal.pcbi.1010006 (PMC9017885; doi:10.1371/journal.pcbi.1010006)
Supplement: Attachment — Submitted filename: GCase_Rebuttal1_TrueFinal.docx [file pcbi.1010006.s008.docx]

The authors would like to thank all reviewers both for their time, and for their helpful feedback for this body of work. We aim to show mechanistic insights about disease-associated mutations observed far from the active sites through our dynamic tools. Therefore, we found suggestions for how to prove the effectiveness of these tools, and to draw comparisons to other contemporary predictive tools to be particularly useful. Furthermore, clarifications of specific, nuanced portions of the work, such as the principle component analysis and Molecular Dynamics simulations allowed us to construct a more comprehensive text that we believe will now appeal to a broader scientific community. Finally, we address the comments regarding our equations, sections of the Introduction, and overall clarity to significantly strengthen the work presented here. We hope that the reviewers find the changes we have made to be satisfactory and, again, greatly appreciate both reviewers’ time and effort taken to assist us in refining this work.

Reviewer #1: In the manuscript submitted by Nicholas J. Ose, et al, with title “Dynamic coupling of residues within proteins as a mechanistic foundation of many enigmatic pathogenic missense variants”, the authors presented a computational study utilizing dynamic coupling index (DCI) and dynamic flexibility index (DFI) to analyze disease related mutations at dynamic allosteric residue coupling (DARC) sites of 144 human enzymes containing 591 pathogenic missense variants. The dynamical correlations among these mutations distal from the enzymatic active sites are systematically evaluated and compared. The manuscript is well organized and presented and should be published after minor revisions to address the following concerns.

1. Figure 5 and its caption are not clear or easy to follow. For example, term “nSVNs” is not defined anywhere in the manuscript.

Thank you for bringing this to our attention. The caption of Figure 5 has been largely rewritten. We took out nSVNs (i.e. non synanomous single nucleotide variants), instead we now use the terms neutral variant and disease associated variant (DAV) in the text. All variants considered in this work are single nucleotide variants in proteins.

2. In the title, it is indicated that Principal component analysis (PCA) was carried out for DFI values of DARC sites. But the PCA seems not to be presented in the section, or at least it is not clear.

We have added a new Methods section detailing the clustering process, and a line clarifying that PCA was used in that process.

Reviewer #2: Ose et al. present a computational study mapping genetic missense mutations to phenotypes. Primary tools include the dynamic coupling index or DCI, which measures the allosteric coupling between two residue sites. The method exerts random external forces on the functional site (e.g., the active site) and monitors responses of other (distal) sites. Residues with larger responses, called DARC sites, are speculated to be more important for the function. The DCI is based on the linear response theory established about 15 years ago and having gained further development in multiple recent studies. Using DCI, the authors have examined disease mutations in the human acid beta-glucosidase and 144 other human enzymes with annotated disease/non-disease mutations. They found that the predicted DARC sites have a higher potential to cause a loss-of-function effect leading to diseases. Disease mutations caused a change of flexibility near the active site and an overall reduction in the dynamic coupling, suggesting the functional relevance of the metric.

Identifying potential disease mutations has a great impact in improving human health. In this paper, the authors try to establish the mechanistic basis for the genotype-phenotype relationship. The question to be addressed is clearly important and some of the correlations are certainly quite interesting.

Incorporating dynamics in predicting disease mutations is not completely new. For example, Ponzoni and Bahar, PNAS 2018. The use of DCI indeed brings some insights, but the significance of the findings is unclear because of the lack of connections with previous studies. For example, how will the authors compare their method with the metrics used in the 2018’s and many other previous studies? Will DCI show a stronger correlation? What about the predictive power (e.g., evaluated using AUC) compared to the current state-of-the-arts? Some discussion on the potential complementary nature of DCI to other sequence-based, structural, or dynamic metrics will also be helpful.

A main argument here is the correlation between DCI and disease mutations. However, a correlation does not guarantee a deterministic role. According to Fig. 3D, there are still plenty of disease sites associated with low DCIs and, more importantly, many non-disease sites associated with high DCIs. The correlation may suggest a functional relevance but does not necessary establish that the metric is deterministic or even a primary factor.

Indeed, the incorporation of dynamics in predicting disease mutation has been done for a long time. Early on (2013 and 2014), we demonstrated the critical role of dynamics in predicting the outcome of missense variants (see (Gerek et al (ref 49 in the manuscript)), and 2014 (Butler et al. (2014 ref 6 in the manuscript))). While we previously showed the importance of protein dynamics in understanding function, particularly the role of flexibility as a differentiating feature in neutral variants vs DAVs (Fig 1 in Gerek et al 2013), here we aim to provide mechanistic insights about often misdiagnosed variants in non-conserved residues positions far from functional (i.e.,active/catalytic) sites.

We agree that a more direct comparison with other metrics will further establish significance. Therefore, we have added new sections in both Results and Methods showing how a neural network trained on our DCI and DFI data performs in comparison to contemporary metrics. In summary, our dynamics-based approach offers a high level of predictive power comparable with other methods, but exceeds these methods when all metrics are evaluated only at highly evolving sites, which tend to be less accurate for evolution- based metrics. We also tested our data using the Rhapsody web server (Ponzoni and Bahar, PNAS 2018). For this data set, we don’t quite see the performance shown in their paper, despite the fact that within their paper, Ponzoni and Bahar’s method obtains around the same accuracy that our method has obtained here. However, there are two distinctions. First, Rhapsody uses *both sequence and dynamic features* to achieve higher accuracy comparable to other methods. Their accuracy drops if only dynamics features are used. Second, their approach is based on Gaussian Network Models, thus Rhapsody solely uses the magnitude of fluctuations; the directionality of fluctuations which also play critical role is not considered. Although the dynamic methods that our groups utilize are similar, DFI and DCI calculations simulate force perturbations in three dimensions, rather than taking the one-dimensional pairwise approach of Rhapsody. To the best of our knowledge, our group is the first to attain such high prediction accuracy using only dynamic information.

The overall flow of the manuscript is clear, but there are some missing details that affect the clarity to some extent. For example:
The description of MD simulations (in Methods) is too short and clearly does not meet the current standard for transparency and reproducibility.

The MD section within Methods has been expanded greatly, including the starting conditions, software used, simulation process and data analysis. The description should now meet reproducibility standards.

It is unclear if the authors performed MD for all the mentioned proteins (including >90 GCases and 144 human enzymes) or just for GCases and used ENMs for other enzymes.

We added clarification in the Introduction, Results, and Methods sections. MD analysis was used for GCase proteins and ENM was used when we analyze 144 enzymes.

The principal component analysis is mentioned but there is no result. Was PCA used in the clustering?

That is correct. To make it clear, we have added the details of our PCA methods for clustering different variants of GCase.

What were the 20-ns simulation windows (p. 20) used for?

We did not use 20 ns time windows. However, 50 ns time windows were used to compute covariance matrices with 25 ns overlapping windows using the last 500 ns of the simulation (i.e. 500 ns to 550ns time frames of the trajectory, then 525 ns to 575 ns …). The DFI and DCI profiles obtained from each covariance matrix are then averaged and the variations of DFI and DCI per positions are calculated to ensure that the dynamic-based profile represents the equilibrium dynamics of well converged simulations. We have added supplemental data that shows the DFI profile of Wild type GCase with variances and also revised the method section to clarify our approach. (Fig S2)

Minor points:
In the abstract, it says 94 GD mutants but in the Results it becomes 200 (p. 5) and then 97 (p. 6). Please check the number and be consistent.

These were referring to different groups of mutants, but including all of them was unnecessary and confusing. We have re-phrased the wording to maintain a consistent value of 94.

Fig. 1. What is the difference between b & c? Are they from different databases? Why are the trends opposite?

Fig. 1 B and C are indeed obtained using different databases. We use the 1k genomes database to provide information on missense variants in general, while we use the Human Gene Mutation Database to provide information on only those missense variants which we know are *pathogenic.* Between Figures 1B and 1C, we can observe that disease mutations are over-abundant at highly conserved positions which was discussed earlier in Kumar et al.2009 (ref 11)

We edited the Introduction and figure 1 caption to try to make this more clear.

P. 4, line 69. ‘…, which adversely impact the prediction accuracy of commonly used methods because they run counter to expectations (Fig 1d).’ This sentence is unclear and needs to be further explained.

We added a citation to a paper, [Kumar](https://doi.org/10.1038/nmeth.2147) et al. (2012) (ref 15 in the text) , which explains how certain prediction methods tend to lose accuracy at highly evolving sites and EvoD trains each rate category separately to increase accuracy at highly evolved sites. We also revised the text accordingly.

P. 8, line 167. ‘DCI measures the coupling of a position’. It should be ‘coupling of two positions.’

Changed to: “coupling of one position to another.” because directionality of the coupling is important.

Fig. 4 legend. It should be ‘DARC sites.’

Fixed.

Not all figure panels are cited in the text.

All figure panels should now be cited in the text.

Reviewer #3: The work presented particularly exciting. The possibility of having a structural explanation for the impact of a point mutation involved in pathology and particularly interesting. This work follows on from other research carried out either on a specific protein or on a large set of data. (State of the art could be deeper).

This work at the border of these two categories and this makes reading the manuscript slightly difficult. I had to reread the entire manuscript several times to be sure what I was reading comma was either the protein involved in Gaucher disease or it was a large number of proteins. I am not sure, moreover, that I have followed everything correctly.

We appreciate your excitement and your feedback. We have revised the manuscript to make these two parts to be more distinct by clarifying the different methods that are used for analysis and keeping the results separate.

Thus figure 1 presented, quickly in the manuscript, relates to a large data set point when Figure 2 it is only on the protein of Gaucher disease. The figures are not analyzed for the specialist. It is difficult to know if the results are significant or not. As an example figure 2A is composed of 1 example, one small region seems different, but not at the point of mutations, but no statistical analysis allow to see it. How is it on the other SNPs?

GCASe is our case study protein that harbors many enigmatic (i.e. allosteric) disease mutations before we expand to the larger set of different human enzymes. In Figure 2, we present a well-studied N370S disease mutation where we have also experimental data, and aim to provide insights how the N370S mutation modifies the dynamics of the functional loop despite the fact that the mutation is not close to this loop, which is a signature of dynamic allostery. Then in Figure 3 we expand this analysis to other variants of GCASE. We have revised our text to make it clear.

And, a general question arises on the use of SNPs associated with this pathology, is it possible to have, thanks to the different projects of 100000 genomes++, all the non-pathological SNPs and then obtain -in fact- exactly the same results. It is a necessary that must be in this paper.

We have constructed a large data set with non-pathological and pathological SNPs as we discuss later in the text and build their accuracy using a machine learning approach.

The work is mainly based on an existing methodology, which must nevertheless be defended in a more rigorous way. It does not seem to be very sensitive. It would be advisable to better integrate its explanation and its critical analysis in the whole of the manuscript.

Thank you for bringing the lack of rigor and critical analysis to our attention. As part of this analysis, we have added new “Neural Network” sections in both Results and Methods showing how training a neural network on our DCI and DFI data performs in comparison to contemporary metrics. In addition, we have added P-values wherever applicable. Please see response to the Reviewer 1

The choice of this enzyme seems to be more related to the distance from the active site as a problem for its function as allosteric questioning, at long range. Figure 3A represents difficulty of reading it is difficult to see what is really relevant from what is not. The black dots and red dots are not separable. The choice of threshold values is not explain. It is difficult to make an opinion.

The threshold value for distance was chosen at 10 Angstroms as a way to ensure that we investigate solely the allosteric (i.e. long distance/ not direct) interactions, meaning substitutions at a distant residue leads to changes in interaction network, thereby indirectly modulating the active site. The top 60% DCI cutoff was chosen to ensure that “DARC” sites had a greater than average response fluctuation.

We would like to have other examples with different shapes and folds.

As discussed above, we have used a diverse set of 144 human enzymes (different folds) harboring neutral and disease associated variants and present that many distal disease mutations exhibits high coupling while neutral ones do not. In this revised version we have also added how using these dynamics features can be used to capture disease associated mutations at distal and highly evolving sites.

Reviewer #4: In this manuscript, the authors compare disease mutations with neutral mutations in human enzymes, in terms of their allosteric dynamic coupling with known enzymatic active sites. The computation analysis is based on a combination of elastic network models and molecular dynamics simulation. The authors conduct both case studies and proteome-wide analyses, concluding that disease mutations tend to disrupt catalytic function through dynamic allosteric coupling with active sites.

On Pages 6-7, in the section entitled "Disease-associated mutations modify dynamics throughout the protein", the authors investigate a single disease mutation N370S in the enzyme GCase, and show that this disease mutation leads to an increase in flexibility (as measured by DFI) within or near loop 1 and/or loop 3. Are the authors suggesting that for the GCase enzyme, disease mutations on average lead to higher DFI values within or near loop 1 and/or loop 3 than neutral mutations? If so, this assertion should be rigorously tested with p-values presented. If not, the authors should clearly describe the conclusions from their analyses.

In general, the manuscript contains numerous general assertions regarding disease versus neutral mutations, only some of which are supported by p-values. The authors should support their general assertions by p-values whenever possible.

P-values have been added wherever applicable

The definition and application of the DCI metric are somewhat confusing. DCI is defined to measure the impact of catalytic site perturbation on a residue under investigation. Here, catalytic site is the cause, and the residue under investigation is the consequence. However, the authors then apply the DCI metric to identify and study allosteric residues, where presumably the residue under investigation is the cause, and the catalytic site is the consequence. What is the rationale for applying DCI in this context, given that the direction of cause and effect seem to be reversed?

This is an excellent questions and one which relates to our results on asymmetry. We have seen that mutation sites which are controlled by the active site, rather than being in control themselves, are more likely to cause disease. Indeed as we presented in our previous work of allosteric binding sites, we observe that perturbation on the binding sites impact the active/catalytic sites as suggested by the review of Campitelli et al. (Ref 25) in the text. However, usually within the field, distal mutations which modulate dynamics are labelled as allosteric mutations. But most importantly, any allosteric interaction has a directionality that breaks symmetry of the response and indeed we observe the same asymmetry in disease variants here.

On Page 10, the first section is entitled "Principal component analysis of DFI aligns with experimentally determined catalytic activity". However, in this section the authors only performed clustering analysis (Fig. 4), but not principal component analysis.

The Principle component analysis was a part of the clustering process. A new section has been added in Methods detailing that process.

On Page 19, Equation (5) contains several errors. The division sign should be "/" rather than "\". In the numerator, the index j should sum over from 1 to N_functional, rather than from N_functional to N_functional. In the denominator, the index beneath the summation symbol should be j rather than i.

Thank you for noting the typos, they have been corrected.

On Page 20, Equation (9), the equation "DCI_asymm = DCI_i - DCI_j" does not make sense and should be fixed and further elaborated.
We have amended the DCI notation to refer to either DCI_ij_ or DCI _ji_. Because DCI is a direction dependent pairwise measurement, it should have notation to indicate both residue pairs and direction. Asymmetry is the difference in DCI when both residues remain the same, but the direction is changed.

It is not clear if the authors have made all data underlying their findings fully available. The authors should try to make their data as fully available as possible. The data can either be provided as supporting information, or deposited to a public repository.

We have made all data available within the supplemental data and also DFI and DCI tools are available at Ozkan GitHub.

Minor comments and typos:

Page 5, Line 98: "wcontaining" -> "containing".
Page 7, Fig. 2 mentions "%DFI profile", but "%DFI" is not defined.
Page 12, Line 273: "sit" -> "site".
Fig. 3a, 3b, and 3d are not referred to in the manuscript text.

All have been fixed.
