# Supplementary material for: Dynamic coupling of residues within proteins as a mechanistic foundation of many enigmatic pathogenic missense variants
Source: PLoS Comput Biol. 2022 Apr 7;18(4):e1010006. doi: 10.1371/journal.pcbi.1010006 (PMC9017885; doi:10.1371/journal.pcbi.1010006)
Supplement: Attachment — Submitted filename: GCase_Rebuttal2.docx [file pcbi.1010006.s009.docx]

The authors would once again like to thank all reviewers for their time and feedback. We are very pleased that the reviewers approve of the work, and are happy to make the minor changes requested here.

Reviewer #1: The authors have addressed all the concerns raised by this reviewer. Now the manuscript should be accepted for publication.

Reviewer #2: The authors have done great work to revise their paper and have addressed all my previous concerns. I only have two additional minor points to mention regarding the new text, which I believe the authors can fix easily:

It would be better to have a brief explanation of the scores (accuracy, recall, etc.) used in the benchmark.

We have added a passage to the methods section describing the scores that we use and how they are calculated.

Fig. 6B, only recalls are shown. Better to show other scores as well for completeness (maybe in the supplemental file).

A new supplemental figure has been added, S3 Fig. This figure shows all scores for either method of finding highly evolving sites. One reason we were reluctant to include such a figure to begin with was that our highly evolving set using MEGA caused particular difficulty for Rhapsody, showing zero or incalculable values for all scores except accuracy.

Reviewer #3: I am particularly and pleasantly surprised by the quality of the responses given to all the reviewers. The authors have taken all the comments into account and wanted to answer all the questions in depth. They did this with great success, which gave me a much better understanding of this work. It deserves to be published as it is and I hope will have the impact it deserves.

Reviewer #4: All comments have been adequately addressed.
